# Changes in gastrointestinal symptoms and food tolerance 6 months following weight loss surgery: associations with dietary changes, weight loss and the surgical procedure

**DOI:** 10.1186/s40608-018-0206-4

**Published:** 2018-12-03

**Authors:** Anne Stine Kvehaugen, Per G. Farup

**Affiliations:** 1 Department of Surgery, Innlandet Hospital Trust, Kyrre Greppsgate 11, 2819 Gjøvik, Norway; 2 Department of Research, Innlandet Hospital Trust, Brumunddal, Norway; 3 Unit for Applied Clinical Research, Department of Clinical and Molecular Medicine, Faculty of Medicine and Health Sciences, Norwegian University of Science and Technology, Trondheim, Norway

**Keywords:** Obesity, Gastrointestinal, Diet, Food tolerance, Weight loss, Weight loss surgery, Roux-en-Y gastric bypass, Vertical sleeve gastrectomy

## Abstract

**Background:**

Gastrointestinal (GI) co-morbidity is common in obese patients, but the effect of weight loss surgery on GI symptoms is incompletely elucidated. The aims of the present study were to explore changes in GI symptoms and food tolerance following weight loss surgery and to study whether such changes were associated with dietary modifications and/or the type of surgical procedure [Roux-en-Y Gastric Bypass (RYGB) versus Vertical Sleeve Gastrectomy (VSG)].

**Methods:**

Participants: Patients with morbid obesity scheduled for weight loss surgery.The patients filled in paper-based questionnaires addressing diet, GI symptoms (bloating, pain, satiety, constipation and diarrhea) and food tolerance/quality of alimentation (satisfaction about current food intake, tolerance to specific foods and frequency of vomiting/regurgitation/reflux) 6 months prior to and 6 months after the surgery. Patients with pre-existing major GI co-morbidity or previous major GI surgery were excluded.

**Results:**

Fifty-four patients (RYGB/VSG: 43/11) were included. Constipation and satiety increased and food tolerance decreased significantly after the surgery (all *p*-values < 0.05). The increase in satiety was significantly more notable after VSG than after RYGB (*p* < 0.05).The increase in satiety also correlated with an overall reduction in food tolerance (rho: -0.488, *p* < 0.01). Divergent changes were seen in the frequency of vomiting/regurgitation/reflux, with a decline after RYGB (*p* = 0.01) and an increase after VSG (*p* = 0.06). Intakes of energy, macronutrients, fiber and fluid decreased significantly after the surgery (all *p*-values < 0.05), but did not correlate with the changes in constipation, satiety or food tolerance (all *p*-values > 0.05). Pre-operatively, total energy intake correlated with bloating and abdominal pain (rho = 0.343 and 0.310 respectively, *p* < 0.05 for both), but these correlations did not persist 6 months after the surgery (rho = 0.065 and 0.054 respectively, *p* > 0.05 for both).

**Conclusion:**

A high caloric intake may explain some of the GI symptoms experienced by non-operated obese patients. The worsening or new-onset of symptoms post-surgery is likely due to anatomical or physiological alterations following surgery. The increase in satiety and the decrease in food tolerance are likely explained by the restrictive nature of the surgeries, as satiety increased more after VSG than after RYGB and correlated with an overall reduction in food tolerance.

## Background

Weight loss surgery is considered an effective tool for the treatment of obesity [1]. In addition to the resultant weight loss, this type of intervention may also ameliorate or reduce associated co-morbidities and risk factors such as hypertension, lipid abnormalities, fasting plasma glucose, HbA1c, metabolic syndrome and type 2 diabetes mellitus [1, 2]. Gastrointestinal (GI) co-morbidity is also common in obese patients [3–5], but less is known about how GI symptoms change pre- to post surgery. A direct relationship between Body Mass Index (BMI) and specific GI symptoms and bowel habits has been shown [6, 7], and it has been speculated that excessive food (nutrient) intake could explain some of these symptoms [3, 6, 7]. Thus, weight reduction per se and/or alterations in diet following weight loss surgery could potentially relieve the GI symptoms experienced by obese patients. On the other hand, weight loss surgeries induce anatomical changes of the GI tract by restricting the gastric volume and, depending on the type of surgery, may also induce some degree of malabsorption. A change in the tolerance to certain foods has also been described as a side effect of these surgeries [8–11]. A broader understanding of the associations between the changes in diet, weight, food tolerance and GI symptoms, as well as the effect of surgery and the type of surgical procedure on GI symptoms and food tolerance is therefore warranted in order to improve the nutritional and medical care of these patients.

Aim of the present study was to explore changes in GI symptoms and food tolerance from 6 months prior- to 6 months after weight loss surgery and to explore whether such changes were associated with alterations in dietary intake, weight loss or the type of surgery [Roux-en-Y Gastric Bypass (RYGB) and Vertical Sleeve Gastrectomy (VSG)].

## Methods

### Patients and study design

The study was performed at the unit for morbid obesity at Innlandet Hospital Trust, Gjøvik, Norway. Patients referred for morbid obesity [BMI ≥ 40 kg/m^2^ or BMI ≥ 35 kg/m^2^ with obesity related complications (e.g. diabetes, hypertension, sleep apnoea, musculoskeletal problems)] were included in a comprehensive study from December 2012 to September 2014. The patients were evaluated for bariatric surgery or non-surgical treatment of obesity. Medical history was taken, physical examination was performed and a blood sample was collected for further analyses. The patients filled in paper-based questionnaires including a case-report form (providing data such as demographics, clinical data and co-morbidities) and a food frequency questionnaire (FFQ) designed to study the usual diet. The FFQ has been prepared and validated by the University of Oslo [12, 13]. Daily intake of food, nutrients and energy was calculated by Department of Nutrition at the University of Oslo by their in-house calculation program (KBS, version 7.3, food database AE-14). The food composition database in the calculation program is based on the official Norwegian food composition table from 2016 [14] and is supplemented with additional food items. The degree of specific GI complaints was assessed with the Gastrointestinal Symptom Rating Scale – Irritable Bowel Syndrome (GSRS-IBS) [15]. The questionnaire contained 13 questions with responses ranging from 1 to 7 (no discomfort at all to very severe discomfort). The questions were clustered into the following dimensions; GSRS-diarrhea, −constipation, −bloating, −pain and –satiety [15], and a mean value for the items in each dimension were calculated. The questionnaire “Quality of alimentation” [16] was used for the evaluation of food tolerance. An overall food tolerance score (FTS): range 1–27, with increasing values representing increased food tolerance, was calculated based on the following responses: 1) an overall assessment of the patient’s satisfaction about food intake [range: 1 (very poor) to 5 (excellent)]; 2) an evaluation of tolerance of eight different types of food [range 0–16; range for each food item: 0 (can’t eat), 1 (can eat with some difficulties/restrictions), 2 (can eat without any difficulties)] and 3) frequency of vomiting/regurgitation/reflux (V/R/R); range: 0 (daily), 2 (three or more times a week), 4 (up to twice a week), 6 (never) [16]. As the terms “reflux” and “regurgitation” are often used interchangeably, our local, non-validated translation of the food tolerance questionnaire included synonyms of both expressions, compared to “regurgitation” only as per original questionnaire.

Patients scheduled for surgery (RYGB or VSG) were included in a longitudinal follow-up study. Repeat measures of the above were performed at the pre-operative day (~ 6 months after the first visit), and in relation to the routine follow-up visits at the hospital (6 weeks, 6 months, 12 months and 24 months after the surgery). Collection of data for research purposes were performed at all, but the 6-week appointment. Dietary data were not obtained at the pre-operative day. The present study compared data obtained from the first visit at the hospital ~ 6 months prior to the surgery, with data obtained from the 6 month follow-up visit after surgery. All patients signed an informed written consent. Patients who completed the FFQs and had data on GI symptoms, food tolerance and anthropometrics at both time points were included. Patients who withdrew their consent for participation were excluded, as were patients with pre-existing major GI co-morbidity or previous major GI surgery.

### Dietary counseling and follow-up

The patients received dietary and lifestyle counseling at enrolment as well as at the follow-up visits. The pre-surgery intervention has been described in more detail previously [17]. The patients also received oral and written information regarding the dietary principles to be followed after the surgery (e.g. selection of foods low in energy, but high in protein and fiber, consuming small portion sizes, chewing the food well, not drinking with meals etc). After the surgery, patients consumed liquid meals for one week and soft/pureed meals the next two weeks (week 2 and 3 after the surgery). From the 4th week, solid foods could be introduced. All patients (without differences between the surgery groups) were prescribed the following daily supplements, starting 10–14 days after the surgery: a multivitamin- and mineral supplement, calcium carbonate with vitamin D (1000 mg/800 IU) and oral iron (corresponding to 100 mg Fe ^2+^ for men and post-menopausal women and 200 mg Fe ^2+^ for pre-menopausal women). The patients also received vitamin B12-injections every three months after the surgery. Compliance with the dietary advices, including the use of supplements, was monitored at the follow-up visits. Blood samples were also collected and based on the test results, adjustments of the supplements were made if indicated.

### Surgical procedures

Standardized laparoscopy with four trocars and a liver retractor. In case of sliding hernia this was repositioned, and a hiatal repair was performed with non-absorbable multifilament 1 suture.

#### VSG

The gastro-colic and -splenic ligaments were divided close to the greater curvature of the stomach, posterior adhesions to the pancreas were released, and the left diaphragmatic crus was exposed. The stomach was divided with linear staplers over a bougiesize of 35 French, from 3 to 4 cm from the pylorus to 0–1 cm from the angle of Hiss. The whole stapler line was inverted with slowly-absorbable 2–0 monofilament suture. The resected part of the stomach was removed.

#### RYGB

The gastric pouch was created with linear staplers, a total line of 100–150 mm. The Roux-en-Y bypass was created with a 50 cm bilio-pancreatic limb (100 cm in patients with type 2 diabetes mellitus) and a 150 cm alimentary limb. The gastro-enterostomy was fashioned with 30–45 mm linear stapler and one row of 2–0 slowly absorbable monofilament suture. The omentum was not divided. The entero-anastomosis was created with a 45 mm linear stapler and closure with 2–0 slowly absorbable monofilament suture. The mesentery at the entero-anastomosis was divided to the vascular arcade (4–7 cm) and the opening in the mesentery and Petersen’s space were both closed with double-rows of single non-absorbable staplers.

### Statistical analyses

Data analyses were performed with IBM SPSS Statistics for Windows, Version 23 (IBM Corp., Armonk, N.Y., USA). For continuous data, comparisons between two independent groups were analyzed with the independent sample t-test if data were normally distributed; otherwise, or in case of ranked data, the non-parametric Mann Whitney U-test was used. Related samples were similarly analyzed with the paired sample t-test or the Wilcoxon Signed Rank test. Categorical data were analyzed with χ^2^statistics. Data were presented as mean with standard deviation (SD), median with 25–75 percentiles or number (n) and percent. Changes from pre- to post-surgery were computed as post-surgery values minus pre-surgery values for all variables. Correlations between variables were tested with Spearman’s correlation (rho). Association analyses were considered explorative.

## Results

### Patients and material

In all, 99 patients underwent either RYGB (*n* = 81) or VSG (*n* = 18) surgery. Of these, 17 patients withdrew their consent for participation at the 6 months follow up and were excluded, as were patients with pre-existing major GI co-morbidity (celiac disease; *n* = 3) or previous major GI surgery (gastric banding; *n* = 2, fundoplication; *n* = 1). Fifty-four (RYGB/VSG: 43/11) of the remaining patients had sufficient data at both time points allowing for comparisons of changes in diet, anthropometrics, food tolerance and/or GI symptoms and were selected for the present study. Age at enrolment was significantly higher [mean (SD): 45.7 (7.74) vs.40.2 (8.36), *p* = 0.001] in the selected study population compared to the patients not included in the study. There were no significant differences between the groups with respect to pre-surgery weight, BMI, GI symptoms or other co-morbidities (all *p*-values > 0.05).

Tables 1 and 2 shows the patient characteristics, GI symptoms, food tolerance measures and the dietary data for the included patients before and 6 months after the surgery, as well as the change in each variable between the two time points. Data on tolerance to individual food items are not displayed in the table. There were significant reductions post-surgery in the tolerance to red meat, rice, pasta and bread (all *P*-values < 0.05), but not to white meat, fish, vegetables and lettuce (all P-values > 0.05).The majority of the intolerant patients responded that they could eat the food with some difficulties (data not shown).

**Table 1:** Changes in patients` characteristics, GI-symptoms and food tolerance from pre- to 6 months post-surgery

	*N*	Pre-surgery	6 months post-surgery	Change ^a^	*P*-value
Age, years	54	45.7 (7.74)	NA	NA	NA
Gender (male/female), n (%)	54	10 (18.5)/44 (81.5)	NA	NA	NA
Type of surgery (RYGB/VSG), n (%)	54	NA	43 (79.6)/11 (20.4)	NA	NA
Weight (kg)	54	120 (110 to 133)	84.8 (78.1 to 95.1)	−34.3 (− 40.3 to −29.0)	< 0.001*
BMI, kg/m^2^	54	41.6 (3.47)	29.8 (3.69)	−11.8 (2.84)	< 0.001*
GSRS-IBS Pain (1–7)	50	1.50 (1.00 to 2.50)	2.00 (1.00 to 2.50)	0.00 (0.00 to 1.00)	0.157
GSRS-IBS Satiety (1–7)	51	1.00 (1.00 to 1.50)	2.50 (1.50 to 3.00)	1.00 (0.00 to 1.50)	< 0.001*
GSRS-IBS Bloating (1–7)	49	1.67 (1.00 to 3.00)	2.00 (1.67 to 3.17)	0.00 (−0.33 to 1.00)	0.094
GSRS-IBS Constipation (1–7)	50	1.00 (1.00 to 2.00)	1.00 (1.00 to 2.50)	0.00 (0.00 to 1.12)	0.030*
GSRS-IBS Diarrhea (1–7)	50	1.25 (1.00 to 1.81)	1.50 (1.00 to 2.25)	0.12 (−0.25 to 0.56)	0.192
FTS (1–27)	53	24.0 (22.0 to 26.0)	22.0 (20.0 to 25.0)	−1.00 (−4.50 to 1.00)	0.006*
Satisfaction of current eating (1–5) ^b^	53	4.00 (4.00 to 5.00)	4.00 (4.00 to 5.00)	0.00 (−1.00 to 1.00)	0.258
Sum score of tolerance to eight individual food items (0–16) ^c^	54	16.0 (14.7 to 16.0)	14.0 (12.0 to 15.2)	−2.00 (−3.25 to 0.00)	< 0.001*
Vomiting/Regurgitation/Reflux (0–2–4-6; daily to never)	54	4.00 (4.00 to 6.00)	6.00 (4.00 to 6.00)	0.00 (−2.00 to 2.00)	0.120

Data are given as median (25–75 percentiles); mean (SD) or n (%). ^a^ The changes were computed as post-surgery values minus pre-surgery values for each patient (paired samples). P-values were obtained by the Wilcoxon Signed Rank Test and the Paired-Sample T-test. GSRS-IBS: Gastrointestinal Symptom Rating Scale-Irritable Bowel Syndrome (range 1–7: Increasing values indicates increasing symptoms). FTS: Food Tolerance Score (range 1–27: Increasing values indicates better tolerance). ^b^ Very unsatisfied to very satisfied. ^c^ Range of each food item: 0 (can’t eat); 1 (can eat with some difficulties); 2 (can eat without any difficulties). * Statistically significant *p* < 0.05

**Table 2:** Changes in daily dietary intakes from pre- to 6 months post-surgery

	*N*	Pre-surgery	6 months post-surgery	Change ^a^	*P*-value
Energy, kJ/kcal	54	9859 (7533 to 12,424)/ 2356 (1800 to 2969)	5298 (4272 to 6541)/ 1266 (1021–1563)	− 4515 (− 6514 to − 2110)/ − 1079 (− 1557 to −504)	< 0.001*
Fat, g	54	89.7 (58.5 to 121)	45.0 (39.7 to 54.3)	−36.0 (− 70.0 to − 15.7)	< 0.001*
Fat, E%	54	35.6 (31.3 to 38.6)	34.3 (28.5 to 38.1)	−1.25 (− 4.80 to 1.92)	0.159
Protein, g	54	103 (81.6 to 127)	65.1 (52.1 to 73.4)	−39.6 (−54.2 to − 24.0)	< 0.001*
Protein, E%	54	18.0 (15.9 to 19.6)	20.1 (18.2 to 22.3)	2.25 (0.05 to 5.47)	< 0.001*
Carbohydrates, g	54	245 (194 to 318)	133 (94.5 to 174)	− 111 (− 158 to −61.5)	< 0.001*
Carbohydrates, E%	54	42.5 (40.1 to 46.7)	42.6 (35.7 to 47.1)	−0.65 (−7.17 to 3.90)	0.247
Sugar, g	54	24.1 (14.3 to 42.5)	11.9 (4.57 to 22.9)	−14.2 (−28.5 to −3.12)	< 0.001*
Sugar, E%	54	4.80 (3.00 to 6.82)	3.70 (1.67 to 6.52)	−1.10 (−3.87 to 1.25)	0.042*
Fiber, g	54	33.8 (9.42)	20.9 (7.91)	−12.9 (9.85)	< 0.001*
Water total, g	54	3602 (2816 to 4756)	2516 (2152 to 3120)	− 1078 (− 1981 to − 148)	< 0.001*
Food and beverages total, g	54	4357 (3389 to 5636)	2995 (2503 to 4190)	− 1090 (− 2332 to −34.1)	< 0.001*

Data are given as median (25–75 percentiles) or mean (SD). ^a^ The changes were computed as post-surgery values minus pre-surgery values for each patient (paired samples). P-values were obtained by the Wilcoxon Signed Rank Test and the Paired-Sample T-test.* Statistically significant *p* < 0.05

### Associations between GI symptoms, food tolerance, diet and anthropometrics

Table 3 shows the correlations between the GI symptoms, food tolerance, weight, BMI, and dietary intakes before the surgery (A) and 6 months after the surgery (B). Correlations between the changes in these variables from pre- to 6 months after the surgery were also examined, and significant correlations were noted for the following variables: A decline in food tolerance was associated with a decline in weight and BMI (rho: 0.283 and 0.298 respectively, *P*-values < 0.05 for both) and with an increase in satiety and abdominal pain (rho: − 0.488 and − 0.362 respectively, P-values < 0.01 for both). A decline in fat intake was associated with a decline in bloating (rho: 0.303, *p* < 0.05), whereas a decline in sugar intake was associated with a decline in abdominal pain (rho: 0.513, *p* < 0.01).

**Table 3:** Correlations between GI symptoms, food tolerance, weight, BMI and dietary intakes

	GSRS-IBS Satiety	GSRS-IBS Pain	GSRS-IBS Bloating	GSRS-IBS Constipation	GSRS-IBS Diarrhea	FTS
Weight, kg	A: − 0.046 B: − 0.253	A: 0.012 B: − 0.200	A: − 0.045 B: − 0.064	A: − 0.010 B: − 0.123	A: 0.080 B: − 0.113	A: 0.041 B: 0.204
BMI, kg/m^2^	A: 0.057 B: − 0.045	A: 0.224 B: − 0.069	A: 0.156 B: 0.015	A: 0.093 B: − 0.187	A: 0.207 B: 0.100	A: 0.025 B: 0.160
Energy, kJ	A: 0.159 B: 0.005	A: 0.310* B: 0.054	A: 0.343* B: 0.065	A: 0.102 B: − 0.102	A: 0.115 B: 0.023	A:-0.163 B: − 0.059
Protein, g	A: 0.152 B: − 0.195	A: 0.190 B: − 0.025	A: 0.289* B: − 0.047	A: − 0.062 B: − 0.099	A: 0.167 B: 0.008	A: − 0.047 B: 0.012
Fat, g	A: 0.057 B: 0.060	A: 0.247 B: 0.031	A: 0.293* B: 0.059	A: 0.019 B: − 0.178	A: 0.215 B: − 0.015	A: − 0.093 B: − 0.077
Carbohydrate, g	A: 0.194 B: 0.097	A: 0.297* B: 0.094	A: 0.312* B: 0.155	A: 0.107 B: 0.006	A: 0.077 B: 0.053	A: − 0.170 B: − 0.009
Sugar, g	A: 0.102 B: 0.118	A: 0.259 B: 0.137	A: 0.251 B: 0.103	A: − 0.029 B: − 0.077	A: 0.066 B: − 0.018	A: − 0.112 B: − 0.081
Fiber, g	A: 0.163 B: − 0.004	A: 0.194 B: − 0.005	A: 0.166 B: 0.010	A: 0.194 B: − 0.057	A: 0.076 B: 0.062	A: −0.177 B: − 0.022
Water total, g	A: 0.176 B: − 0.081	A: 0.240 B: − 0.041	A: 0.186 B: − 0.017	A: − 0.010 B: 0.047	A: 0.167 B: − 0.026	A: −0.214 B: 0.092
Food and beverages total, g	A: 0.168 B: −0.031	A: 0.282* B: 0.035	A: 0.247 B: 0.131	A: 0.026 B: 0.072	A: 0.178 B: 0.073	A: −0.216 B: 0.099
FTS	A: −0.298* B: − 0.358**	A: − 0.376** B: − 0.399**	A: −0.304* B: − 0.206	A: − 0.353** B: 0.116	A: − 0.335* B: − 0.016	A: 1.000 B: 1.000

Correlations are given as Spearman’s rho: A: Pre-surgery; B: 6 months post-surgery. * Statistically significant *p* < 0.05. ** Statistically significant *p* ≤ 0.01

GSRS-IBS: Gastrointestinal Symptom Rating Scale-Irritable Bowel Syndrome (Increasing values indicates increasing symptoms). FTS: Food Tolerance Score (Increasing values indicates better tolerance)

### RYGB vs. VSG

**Fig. 1:**
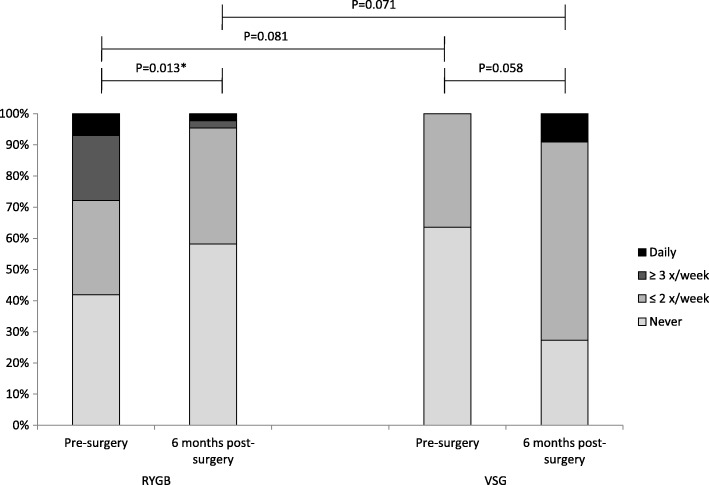
The figure shows the frequency (%) of vomiting/regurgitation/reflux pre-and post surgery for each of the surgery groups (RYGB: Roux-en-Y Gastric Bypass and VSG: Vertical Sleeve Gastrectomy). *P*-values are given for the within-group changes (pre-to post surgery) as well as comparisons between the two surgery groups at each time point. *Statistically significant *p* < 0.05

Patient characteristics and dietary data were similar between the two surgery groups at enrolment in the study (all *P*-values > 0.05) and the variables did not change significantly differently between the groups (all P-values > 0.05). The exception was BMI, which was significantly higher in the RYGB group compared to the VSG group [42.1 (3.42) vs. 39.7 (3.13), *P* = 0.039]. BMI remained higher, but not significantly, also at the 6 month follow-up [30.2 (3.67) vs. 28.1 (3.42), *P* = 0.089]. Table 4 shows the changes in GI symptoms and the FTS from pre-to post surgery in each of the surgery groups, as well as comparisons between the groups. GSRS-IBS satiety increased significantly in both groups, but significantly more so in the VSG – than the RYGB-group. Neither the FTS (Table 4), nor its specific items (data not shown), differed significantly between the groups at any time point. However, between-group comparisons of the changes from pre-to post-surgery, revealed significant differences in the changes of the V/R/R score (*P* = 0.009): From pre- to post-surgery, the V/R/R score increased significantly in the RYGB group and declined in the VSG group; the differences in the changes were in disfavour of the VSG group (Fig. 1).

**Table 4:** Gastrointestinal symptoms and food tolerance before and 6 months after the surgery in the two surgery groups

	Pre-surgery		6 months post-surgery	
	RYGB	VSG	RYGB	VSG
GSRS-IBS-Pain (1–7)	1.50 (1.00 to 2.50)	1.00 (1.00 to 2.00)	2.00 (1.00 to 2.50)	2.50 (1.00 to 3.00) ^c*^
GSRS-IBS-Satiety (1–7)	1.00 (1.00 to 1.50)	1.00 (1.00 to 1.00)	2.00 (1.50 to 3.00)	3.00 (2.50 to 4.00) ^a*, b*, c*, d*^
GSRS-IBS-Bloating (1–7)	2.00 (1.25 to 3.08)	1.67 (1.00 to 2.33)	2.33 (1.67 to 3.33)	1.67 (1.33 to 3.00)
GSRS-IBS-Constipation (1–7)	1.00 (1.00 to 2.00)	1.00 (1.00 to 1.00)	1.00 (1.00 to 2.50)	1.00 (1.00 to 3.50) ^c*^
GSRS-IBS-Diarrhea (1–7)	1.25 (1.00 to 2.00)	1.00 (1.00 to 1.50) ^a*^	1.75 (1.25 to 2.25)	1.25 (1.00 to 1.75)
FTS (1–27)	24.0 (22.0 to 26.0)	25.5 (23.0 to 27.0)	23.0 (20.0 to 26.0)	20.0 (19.0 to 24.2) ^c*^

Data are given as median (25–75 percentiles). Mann-Whitney U-test (between group comparisons); Wilcoxon Signed Rank Test (within group comparison of changes). * Statistically significant *p* < 0.05: ^a^ RYGB vs. VSG; ^b^ Changes pre-to post-surgery within group (RYGB); ^c^ Changes pre-to post-surgery within group (VSG); ^d^ between-group comparisons (RYGB vs. VSG) of the changes pre-to post-surgery. GSRS-IBS: Gastrointestinal Symptom Rating Scale-Irritable Bowel Syndrome (range 1–7: Increasing values indicates increasing symptoms). FTS: Food Tolerance Score (range 1–27: Increasing values indicates better tolerance)

## Discussion

### Changes in GI symptoms and food tolerance

Of five GI symptom dimensions investigated in the present study; GSRS-IBS pain, −bloating, −constipation, −diarrhea and -satiety, a significant increase post-operative was seen for satiety and constipation. In general, increased satiety is considered a desirable effect of these surgeries, as it may aid in the restriction of food intake and thereby facilitate weight loss. Suggested mechanisms include restriction of gastric volume and modifications of GI hormones involved in the regulation of hunger and satiety [18]. There is a lack of tools developed specifically to address GI symptoms in obese patients before and after weight loss surgeries. Thus, some of the questionnaire items, such as satiety, may have different connotations in patients with functional- or organic GI disorders compared to patients after weight loss surgery. It is therefore not known if the increased satiety was perceived as discomfort, equivalent to what has been reported in patients with IBS [15]. In line with previous studies [8, 10, 11, 16, 19, 20], food intolerance was a common complication after the surgery. The finding of a correlation between increased satiety and reduced food tolerance in the present study implies that the increased satiety was in part an unfavorable outcome. However, satisfaction about current food intake was rated high (25 and 75 percentiles corresponding to “good” and “excellent”) both prior to the surgery and at the 6 month follow-up.

Other studies on GI symptoms following weight-loss surgery have provided mixed findings [8, 10, 21–30]. This may partly relate to differences in the study design (e.g. cross-sectional vs. longitudinal), different tools used for the evaluation of GI symptoms and time elapsed since the surgery. Among the longitudinal studies, Ballem N. et al. found a trend towards decreased passing of stools 1–5 years after RYGB [22]. Other symptoms improved or remained unchanged [22]. Afshar S. et al. also found a reduced frequency of bowel motions and a change towards firmer stools for a median of 6.4 months following RYGB and VSG [21]. These results are in agreement with the present findings. In contrast, Potoczna N. et al. found that flatulence and diarrhea increased a median of 2.1 years following RYGB surgery [29]. Others reported an improvement in several GI symptoms 3–12 months post-operatively [23, 28, 30].

### Associations between GI symptoms, food tolerance, weight, BMI and dietary intake

There is a lack of studies examining associations between diet and GI symptoms before and after weight loss surgery. In the present study, total energy intake was reduced by ~ 46% from pre-to post-surgery, with similar reductions in each of the energy-yielding nutrients. There were no associations either between the reduced energy intakes and changes in GI symptoms, or between weight- or BMI loss and changes in GI symptoms, including satiety. The reduction in food tolerance correlated with the reduction in weight and BMI, but not with the reduction in total energy intake or specific nutrients. However, the impact of food intolerance on the dietary intake in the earlier post-operative period was not examined. In general, food tolerance improves with time [19, 20] and it has been shown that patients can establish a diet of nutritional value that is close to that of the general population after a few months of adaption [11].

Along with the reduction in total energy intake, dietary fiber was reduced by ~ 38% and total water/fluid intake was reduced by ~ 30%. The reduction in dietary fiber along with increased symptoms of constipation pre-to post surgery is in line with the study by Afshar S. et al. [21]. However, neither the change in fiber intake, nor the change in water intake, correlated with the change in constipation in the present study. The use of dietary supplements, such as iron, could be another cause of constipation in the post-bariatric patient [31].

Before the surgery, total energy intake correlated significantly with bloating and abdominal pain. Bloating correlated with all of the individual energy yielding nutrients, whereas pain correlated significantly with carbohydrates only. Abdominal pain also correlated with the total amount of food and beverages ingested. Different mechanisms may explain these associations: Increased oral intake could result in gastric distension and abdominal pain [23]. Of the energy yielding nutrients, fat may delay intestinal gas transport and result in abdominal distension [32]. Moreover, total energy intake [33], as well as a “western”/“fast food” dietary pattern characterized by fatty and sugary products, has been associated with the Irritable Bowel Syndrome [34]. The association between carbohydrates and symptoms could possibly also be due to a high load of poorly absorbable and easily fermentable short-chain carbohydrates [35]. Specific dietary patterns were however not explored in the present study. Despite the significant reductions in total food-, energy- and macronutrient intakes, the symptom scores for pain and bloating remained stable after the surgery. Also, at the 6 months follow-up, the symptoms were no longer associated with the dietary intakes. In all, these results suggest that surgically induced changes were responsible for the maintenance, increase or new-onset of symptoms. Factors such as meal size, speed of eating and degree of mastication could also play a role for the degree of GI symptoms and food intolerance after the surgery [36, 37], but were not evaluated in the present study. The increase in constipation and satiety post-surgery may as well have induced more symptoms of bloating and/or pain. On the other hand, significant correlations were seen between the change in dietary fat intake and change in bloating, and between the change in sugar intake and change in abdominal pain (the greater the reductions in these macronutrients, the greater the reduction in symptoms), suggesting that at least a sub-group of patients benefited from the dietary modifications. Relevant to this finding is the study by Petereit R. et al., which found that GI symptoms improved post-RYGB along with changes in eating behavior (increased cognitive restraint and decreased uncontrolled and emotional eating) [28]. Changes in eating behavior were however not addressed in the present study. Moreover, a recently published study by our group reported a reduction in overall symptoms, diarrhea and bloating during the pre-operative conservative weight loss intervention [17]. A limitation is that dietary data was recorded only once during that time period, and correlations between the changes in diet and changes in bowel symptoms could therefore not be performed [17].

### RYGB vs. VSG

There is a lack of longitudinal studies comparing changes in specific GI symptoms between patients that underwent RYGB and patients that underwent VSG. Some studies found that flatulence and diarrhea were more common after mixed/malabsorptive procedures, whereas constipation was more common after purely restrictive procedures [24, 29]. Such differences between the surgery groups were not seen in the present study. However, analyses of within-group changes showed a significant worsening of constipation in the VSG group, but not in the RYGB group. This was due to a non-significantly lower symptom score at baseline and a non-significantly higher symptom score at follow-up in the VSG group compared to the RYGB group. Similar results were obtained for the change in the score for abdominal pain and food tolerance. Thus, procedure specific effects on GI symptoms cannot be excluded, and the present results highlight the need for paired data when evaluating outcomes after the surgery.

The dimension “satiety”, which increased significantly after both procedures, was also more notable after VSG than after RYGB. We are not aware of other studies that used the GSRS-IBS questionnaire after weight loss surgery. Satiety is unique to this questionnaire and direct comparisons to other studies were not possible. The GSRS-IBS questionnaire addresses the degree of satiety after food intake in general (feeling full shortly after meal initiation, as well as feeling full even for a long period of time after stopped eating) [15]. By the use of Visual Analogue Scales for satiety pre-and post-operatively, Valderas JP et al. [18] and Yousseif A et al. [38] found that RYGB and VSG surgeries produced a significant [18, 38] and similar [38] (between-group comparisons of the changes were not reported by Valderas et al.) increase in postprandial satiety. However, these studies measured nutrient stimulated satiety/fullness perception after a standard liquid test meal (AUC _0–180_). Time elapsed since the surgery was 8 weeks [18], 6 and 12 weeks [38] respectively. In the present study, patients were evaluated 6 months post-surgery, i.e. at a time point when solid foods had been introduced. Thus, differences in the perception of satiety between the two surgery groups may perhaps depend on the texture of the food. However, more research in this area is needed.

Results from the food tolerance questionnaire showed that the frequency of V/R/R was significantly improved in the RYGB group and marginally worsened in the VSG group. Pre-operatively, none of the patients scheduled for VSG presented with frequent or daily symptoms of V/R/R compared to approximately 1/3 in the RYGB group. Given current evidence which suggests that gastroesophageal reflux is attenuated after RYGB [39], the presence of this symptom is usually considered when patients are counseled to either procedure. This indicates that reflux/regurgitation rather than vomiting was the predominant symptom, with improvement post-RYGB. Although the literature has been ambivalent; several studies have also shown a worsening or new onset of reflux symptoms after VSG [40]. Moreover, results from two recent randomized clinical trials, with a follow-up period of 5 years, both found that severe reflux was a main reason for reoperation in the VSG group [41, 42].

### Strengths and limitations

The small sample size, in particular the low number of VSG patients, reduced the statistical power of the study. The presence of type I statistical errors can also not be excluded as the study was considered explorative and adjustments for multiple tests were not performed. Strength of the study was the longitudinal design, with paired samples and comparisons of changes between the surgery groups. The use of validated instruments for the measurement of GI symptoms, food intolerance and dietary intakes also represents strengths of the study. A limitation is that the GSRS-IBS, unlike the original GSRS from which it is derived, does not contain questions about upper GI symptoms. This limitation was partly compensated for by the use of the food tolerance questionnaire, which addresses the frequency of V/R/R.

## Conclusion

Constipation and satiety increased and food tolerance decreased 6 months after the surgery. The changes were not associated with the alterations in diet, such as the reduced intakes of energy, fiber and fluid, but food intolerance was associated with total weight loss. Satiety increased more after the purely restrictive procedure (VSG) than after the mixed procedure (RYGB) and satiety correlated with an overall reduction in food tolerance. The frequency of V/R/R also increased marginally after VSG, but declined significantly after RYGB. Abdominal pain and bloating correlated with total energy intake before the surgery, but not 6 months after the surgery. Whether alterations in diet such as increased intakes of energy dense foods could result in more symptoms with longer duration of follow-up remain to be explored.

## Abbreviations

BMI: Body Mass Index
FFQ: Food Frequency Questionnaire
FTS: Food Tolerance Score
GI: Gastrointestinal
GSRS-IBS: Gastrointestinal Symptom Rating Scale-Irritable Bowel Syndrome
RYGB: Roux-en-Y Gastric Bypass
V/R/R: Vomiting/Regurgitation/Reflux
VSG: Vertical Sleeve Gastrectomy
